# Whole-genome sequencing of multiple myeloma reveals oncogenic pathways are targeted somatically through multiple mechanisms

**DOI:** 10.1038/s41375-018-0103-3

**Published:** 2018-04-09

**Authors:** Phuc H. Hoang, Sara E. Dobbins, Alex J. Cornish, Daniel Chubb, Philip J. Law, Martin Kaiser, Richard S. Houlston

**Affiliations:** 1 Division of Genetics and Epidemiology, The Institute of Cancer Research, London, UK; 2 Division of Molecular Pathology, The Institute of Cancer Research, London, UK

## Abstract

Multiple myeloma (MM) is a biologically heterogeneous malignancy, however, the mechanisms underlying this complexity are incompletely understood. We report an analysis of the whole-genome sequencing of 765 MM patients from CoMMpass. By employing promoter capture Hi-C in naïve B-cells, we identify *cis*-regulatory elements (CREs) that represent a highly enriched subset of the non-coding genome in which to search for driver mutations. We identify regulatory regions whose mutation significantly alters the expression of genes as candidate non-coding drivers, including copy number variation (CNV) at CREs of *MYC* and single-nucleotide variants (SNVs) in a *PAX5* enhancer. To better inform the interplay between non-coding driver mutations with other driver mechanisms, and their respective roles in oncogenic pathways, we extended our analysis identifying coding drivers in 40 genes, including 11 novel candidates. We demonstrate the same pathways can be targeted by coding and non-coding mutations; exemplified by *IRF4* and *PRDM1*, along with *BCL6* and *PAX5*, genes that are central to plasma cell differentiation. This study reveals new insights into the complex genetic alterations driving MM development and an enhanced understanding of oncogenic pathways.

## Introduction

Multiple myeloma (MM) is a clinically and biologically heterogeneous malignancy characterized by the infiltration of clonal plasma cells in the bone marrow [1–5]. Despite recent advances in its treatment, MM essentially remains an incurable malignancy with relapse characterized by progressively resistant mutational profiles. Myeloma cells are typified by recurrent chromosomal aberrations, a number of which are associated with poor prognosis, notably t(4;14), t(14;16), t(14;20), 17p deletion, and gain of 1q [1]. However, so far the molecular mechanisms responsible for the initiation and heterogeneous evolution of MM remain largely unknown. The identification of driver mutations as distinguished from passenger mutations is therefore fundamental to understanding MM oncogenesis and its response to therapy.

The search for driver mutations in MM has so far been focused on the protein-coding components of the genome driven by the large-scale sequencing of MM exomes [2–4]. With affordable whole-genome sequencing (WGS), it is now apposite to systematically examine non-coding regions of cancer genomes for driver mutations.

**Fig. 1:**
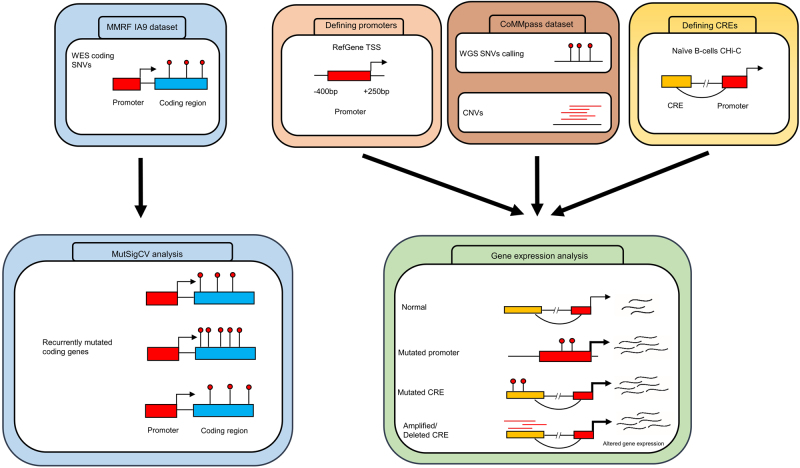
Schematic analysis of the workflow. WES whole-exome sequencing, WGS whole-genome sequencing, SNV single-nucleotide variant, TSS transcription start site, CNV copy number variant, CRE *cis-*regulatory element

Although mutation recurrence is an indicator of positive selection in tumors, the sheer size of the non-coding genome imposes a high statistical burden on robustly establishing recurrent mutations. *Cis*-regulatory elements (CREs) and promoters modulating gene expression represent a highly enriched subset of the non-coding genome in which to search for driver mutations. Therefore, to both reduce the search space and segment the genome into functional blocks, we have utilized information from promoter capture Hi-C (CHi-C) in naïve B-cells [6] and transcription start site (TSS) proximity in an analysis of WGS data on 765 MM tumors. By linking these data to gene expression (Fig. 1), we identified recurrently mutated non-coding regulatory regions. Integrating these data with information on coding drivers together with structural variants and mutational signatures, we have been able to provide a more comprehensive mutational landscape of MM, thereby enhancing our understanding of the oncogenic pathways and mechanisms relevant to MM biology.

## Results

We analyzed whole-exome sequencing (WES) and WGS data of 804 and 765 MM tumor-normal pairs, respectively, generated by The Relating Clinical Outcomes in Multiple Myeloma to Personal Assessment of Genetic Profile Study (CoMMpass, IA9 release [7]). The median age of patients at diagnosis was 64 years (range 31–93) and only baseline newly diagnosed bone marrow samples were considered. The frequency of MM translocation subgroups in the CoMMpass series is similar to that reported in unselected patients [1] (Supplementary Table 1). The median exonic mutation rate across all tumor samples was 1.95 mutations/Mb consistent with published literature [1, 8], with t(14;16) MM displaying the highest mutation rate [9] (*P* = 2.2 × 10^−6^, Wilcoxon rank-sum test; Supplementary Table 1). Although the low-coverage WGS data (average 6–12×) was not primarily produced for mutational analysis, we estimated an average sensitivity of 20% to detect clonal single-nucleotide variants (SNVs) based on comparisons between paired WGS and WES (average 120–150×) data available for 734 samples. A global whole-genome comparison with previously published mutation rates in MM [1, 8] suggests up to 35% sensitivity. Given this limitation, we therefore expect our analysis to provide insights into mostly clonal mutation associated with early events underlying tumorigenesis [10].

### Recurrently mutated non-coding regulatory regions

After quality control and filtering of WGS data, we identified 71,573 SNVs across all tumors. Recurrently mutated regions were identified as those containing highly clustered mutations and a greater number of mutations than that expected given the background mutation rate (see Materials and methods section). To identify somatic mutations in the non-coding regulatory regions, we defined 28,629 regions associated with 23,635 genes as promoters [11]. We identified promoters associated with 34 target genes as recurrently mutated (*Q* < 0.05, Supplementary Table 2). Using promoter CHi-C in naïve B-cells [6], we then defined 79,894 fragments containing putative CREs identifying 221,380 unique significant interactions with promoters. These CRE fragments (median size 2 kb with median linear distance to respective interacting promoter of 300 kb) constituted 15% of the genome and were enriched for ATAC-seq accessibility and regulatory histone marks [6]. We identified 114 recurrently mutated CRE regions, interacting with the promoters of 271 genes (*Q* < 0.05, Supplementary Table 3). These genes were over-represented for pathways associated with cell adhesion (*P* = 4.4 × 10^−4^), inflammatory response (*P* = 5.6 × 10^−4^), nuclear factor κB-inducing kinase/nuclear factor κB (NIK/NF-κB) signaling (*P* = 1.7 × 10^−2^), regulation of B-cell activation (*P* = 3.6 × 10^−2^), and B-cell differentiation (*P* = 4.7 × 10^−2^), including *PAX5* and *BCL6*. (Supplementary Table 4).

### Effect of regulatory SNVs on gene expression

To identify non-coding driver mutations in regulatory regions, we compared the expression levels of respective target genes in mutated and non-mutated tumors. Tumors having copy number changes overlapping either the regulatory region or target gene were excluded from the analysis.

We identified recurrent mutation of the *NBPF1* promoter (20 tumors, *Q* = 1.3 × 10^−15^); these mutations were associated with increased *NBPF1* expression (*Q* = 7.9 × 10^−4^, 1.7-fold; Supplementary Fig. 1). *NBPF1* belongs to the neuroblastoma breakpoint family, members of which have been observed to be overexpressed in sarcomas [12] and non-small-cell lung cancer [13]. *NBPF1* is directly regulated by NF-κB [14], whose signaling pathway is recurrently affected in MM, suggesting the relevance of this novel candidate in MM development.

**Fig. 2:**
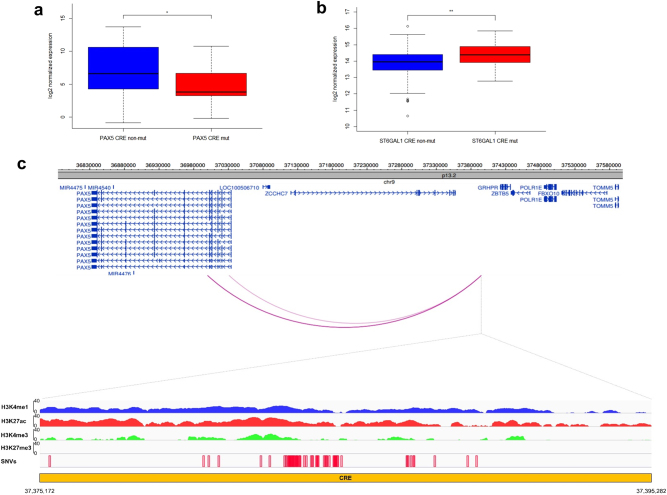
SNVs at* cis*-regulatory elements affect gene expression in multiple myeloma. Mutations in the CRE significantly alter **a** *PAX5* (*n* = 197 versus *n* = 13) and **b** *ST6GAL1* (*n* = 315 versus *n* = 15) expression. Difference in expression was assessed pairwise by negative binomial test. *Q < 0.1, **Q < 0.05. The hinges of the boxplot indicate the first and third quartile range. **c** Chromatin looping interactions between *PAX5* promoter and differentially expressed CRE. Also shown are the ChIP-seq signals and relative positions of SNVs

Six recurrently mutated CREs associated with differential expression of their respective target genes were identified (*PAX5, ST6GAL1, CALCB, COBLL1, HOXB3*, and *ATP13A2*), four annotated by epigenetic marks indicative of active enhancers (*Q* < 0.1, Figs. 2a, b, Supplementary Fig. 2, Supplementary Table 5). The *PAX5* CRE (71 clustered mutations across 55 tumors, 7% of all tumors) maps 3-kb downstream of the *PAX5* chronic lymphocytic leukemia (CLL) enhancer [15] (Fig. 2c). The 4.6-fold reduced expression associated with CRE mutation is consistent with *PAX5* functioning as a tumor suppressor in MM, as in other B-cell malignancies [15–17]. This CRE forms part of a cluster of 12 recurrently mutated CRE fragments interacting with the *PAX5* promoter (Supplementary Table 3). Although 28% (212/765) of tumors harbored mutations in at least one of these *PAX5* CREs, the mutations were not always associated with a significant change in *PAX5* expression. Five CREs, interacting with the *ST6GAL1* promoter, were recurrently mutated in a total of 8% (64/765) of samples. Although the mutated CREs showed an overall consistent trend of association between mutation and upregulation of *ST6GAL1*, only one CRE was significantly associated with increased gene expression (3% of samples, *Q* = 0.036, 1.4-fold upregulation, Fig. 2b, Supplementary Table 5). *ST6GAL1*, which primarily generates α2,6 linked sialic acids on *N-*glycans, is overexpressed in multiple cancers [18] and the increased expression may contribute to aberrant immunoglobulin-G glycosylation seen in MM development [19, 20].

Mutations of the *COBLL1* CRE were associated with increased gene expression. *COBLL1* plays a role in NF-κB pathway activation, is important for normal hematopoiesis [21] and is upregulated in MM [22]. Conversely, mutations in the *HOXB3* CRE were associated with reduced expression, consistent with *HOXB3* acting as a tumor suppressor in MM, as in acute myeloid leukemia [23].

By restricting analysis to subgroups of MM, we identified a CRE interacting with the *TPRG1* promoter as recurrently mutated, resulting in significant differential gene expression in HD and *MYC-*translocation MM (Supplementary Table 6a**)**. Although mutated in only 2% of HD (9/423) and 3% (3/109) of *MYC-*translocation samples, these were associated with 6.3-fold and 3.6-fold upregulation in gene expression respectively (based on 4/118 and 3/34 tumors, respectively; Supplementary Fig. 3). We also identified a relative paucity of mutations in regulatory regions of *PAX5* in t(11:14) MM (*P* = 2.7 × 10^−3^, Supplementary Table 6b). Intriguingly, as this subgroup is enriched for coding mutations in *IRF4*, it suggests complementary genomic alteration impacting on the plasma cell differentiation pathway in MM **(**Supplementary Table 7**)**.

### Copy number variations at CREs regulate gene expression

**Fig. 3:**
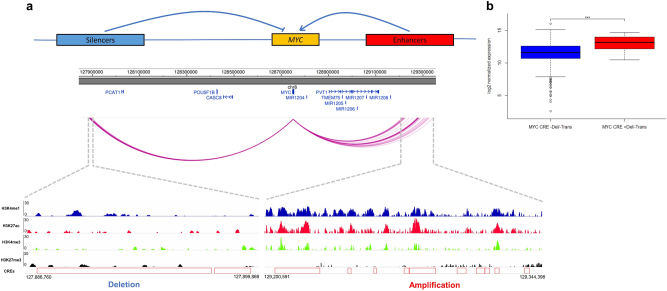
Copy number variations at *cis*-regulatory elements affect *MYC* gene expression in multiple myeloma. **a** Upper panel shows *MYC* gene expression may be regulated by CREs; CNVs at either the upstream putative silencers or downstream putative enhancers causing upregulation of *MYC*. Middle panel shows chromatin looping interactions between *MYC* promoter and CREs. Lower panel details ChIP-seq signals and relative positions of CNVs at these CREs in naïve B-cells. **b** CNV status at CREs and *MYC* expression. Difference in expression was assessed pairwise between samples with different CNVs status and the same translocation status by negative binomial test. ***P < 0.01. Trans translocation, Del deletion. From left to right, *n* = 345, *n* = 9, respectively. The hinges of the boxplot indicate the first and third quartile range

We examined the relationship between copy number variation (CNV) at CREs and expression of interacting genes, excluding CNVs that contained both the CRE and its respective target gene from the analysis. The *MYC* promoter showed both upstream and downstream interactions with 69 CREs; 24 were amplified across 51 tumors and these had significantly higher *MYC* expression (*Q* < 0.05, Supplementary Table 8a). These 24 CRE regions clustered within a 110-kb region forming 10 non-contiguous regions 500-kb downstream of *MYC* annotated by epigenetic marks indicative of active enhancers (i.e., overlapping with strong signals of H3K4me1, H3K27ac, and weak signals of repressive H3K27me3) (Fig. 3a). Five CRE regions upstream of *MYC* interacting with *MYC* promoter were deleted in 10 tumors (distinct from the 51 tumors with CREs amplified), which were associated with higher *MYC* expression (*Q* < 0.1, Supplementary Table 8b). These CREs, clustered within a 13-kb region, 850-kb upstream of *MYC*, form two non-contiguous regions with weaker signals for H3K4me1, H3K4me3, and H3K27ac, and stronger signals for repressive mark H3K27me3, consistent with putative silencers of *MYC* (Fig. 3a).

As *MYC* is translocated in 15–20% of newly diagnosed MM [1] (14% of CoMMpass samples, Supplementary Table 1), we examined the possibility that upregulation of *MYC* expression associated with CRE CNVs might be the consequence of translocation of *MYC* to proximal super-enhancers. We defined a broader set of 209 samples with putative *MYC* translocations (24% of total tumors) and identified that the 51 samples with amplified CREs are indeed highly enriched for translocations (34/51, *P* *=* 1.2 × 10^-11^, Fisher’s exact test), with the breakpoints mapping to the region of amplification. The deletions at CREs were not, however, enriched for translocations (1/10, *P* = 0.9) and in *MYC* translocation negative cases the CNVs at *MYC* CREs were still associated with significantly increased *MYC* expression (Fig. 3b, *P* = 8.6 × 10^-3^, 2.3-fold).

We identified six other novel candidate genes whose expression was significantly altered by CNVs at respective interacting CREs: *PACS2*, *TEX22*, *KDM3B*, *RAB36*, *PLD4*, and *SP110* (Supplementary Fig. 4, Supplementary Table 8). Although each of the respective CREs were annotated by epigenetic marks indicative of functional regulatory regions, these genes reside close to regions of common structural variation, making interpretation of their specific relevance problematic.

### Pathways targeted by both coding and non-coding mutation

To better inform the interplay between non-coding driver mutations with other driver mechanisms, and their respective roles in oncogenic pathways, we extended our analysis of the CoMMpass dataset. We systematically cataloged coding SNVs, copy number and structural variants (Supplementary Note, Supplementary Fig. 5-6, Supplementary Table 9-14). Frequencies of chromosomal copy number alterations and structural variants were comparable to that previously reported (Supplementary Note, Supplementary Fig. 5-6, Supplementary Table 9-10). Applying MutSigCV [24] to variants identified in WES data on 804 patients, we identified 33 genes significantly mutated (*Q* < 0.05, Supplementary Table 11); 16 documented to be recurrently mutated (*KRAS*, *NRAS*, *HIST1H1E*, *MAX*, *SP140*, *RASA2*, *FCF1*, *DIS3*, *BRAF*, *TP53*, *SAMHD1*, *TRAF3*, *PRKD2*, *TGDS*, *CYLD*, and *RB1*; Supplementary Table 13) [1–4, 7] 12 previously reported, albeit not significantly (*PTPN11*, *DNAH5*, *MYH2*, *BMP2K*, *ZNF208*, *RPL10*, *FBXO4*, *OR5M1*, *PTH2*, *CELA1*, *OR9G1*, and T*NFSF12*) [2–4, 25–27] and five novel (*TBC1D29*, *RPS3A*, *BAX*, *C8orf86*, and *FTL*; Supplementary Table 11).

**Fig. 4:**
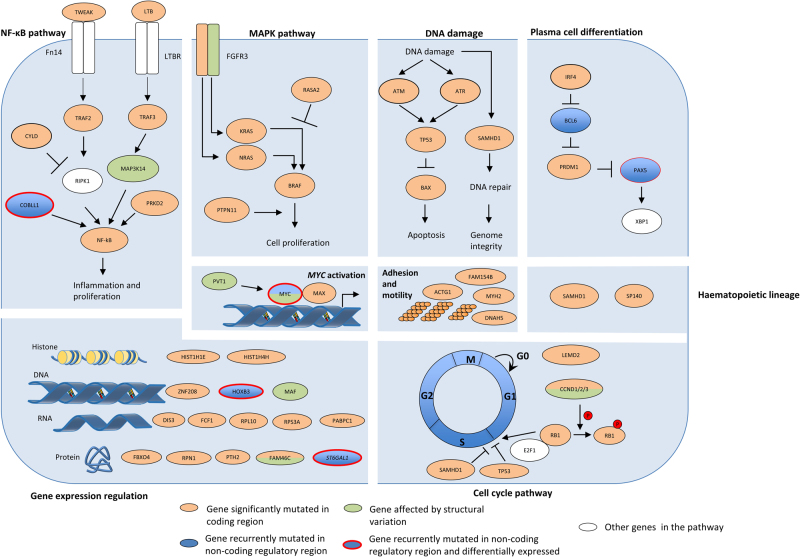
Several key pathways in multiple myeloma are disrupted by a range of mechanisms. Adapted from Manier et al. [1] and Kumar et al. [60]

We identified pathways targeted by coding and non-coding mutations using the Reactome pathway tool [28]. These included mitogen-activated protein kinase (MAPK) signaling, NF-κB signaling, cytokine signaling, G protein-coupled receptors signaling, transcriptional and post-translational expression regulation, hematopoietic development, DNA damage, and apoptosis (*Q* < 0.05, Supplementary Table 15). Many of the genes in these pathways are targeted by both coding and non-coding drivers (Table 1, Fig. 4), exemplified by *IRF4* and *PRDM1*, along with *BCL6* and *PAX5*, genes central to plasma cell differentiation [1].

**Table 1:** Summary of novel findings

Novel genes disrupted in coding regions		Novel genes disrupted by mutations in non-coding regions		
Genes disrupted by structural variants	Genes disrupted by SNVs and indels	Promoters disrupted by SNVs	CREs disrupted by SNVs	CREs disrupted by CNVs
*CD96*	*BAX*	*NBPF1*	*CALCB*	*MYC*
*PRDM1*	*C8orf86*		*COBLL1*	*PLD4* ^a^
*FBXW7*	*FAM154B*		*HOXB3*	*KDM3B* ^a^
*MAP3K14*	*FTL*		*ST6GAL1*	*SP110* ^a^
*CCND2*	*HIST1H4H*		*PAX5*	*RAB36* ^a^
	*LEMD2*		*ATP13A2*	*PACS2* ^a^
	*PABPC1*		*TPRG1*	*TEX22* ^a^
	*RPN1*			
	*RPS3A*			
	*SGPP1*			
	*TBC1D29*			

^a^These genes reside close to regions of common structural variation, making interpretation of their specific relevance problematic

### Mutational signatures

To gain insight into the etiological basis of MM mutations, we analyzed mutational signatures [8]. Mutational signature 2 (C > T/G in TC dinucleotide motif), a consequence of the activity of the APOBEC family of cytidine deaminases [8], associated with poor prognosis [8, 9], was seen in 30% (230/765) of tumors (Supplementary Table 16) and associated with coding mutations in *DNAH5* (*P* = 8.8 × 10^−7^), *SAMHD1* (*P* = 7.2 × 10^−4^), *TP53* (*P* = 9.3 × 10^−3^), and *BRAF* (*P* = 3.7 × 10^−2^). This signature was primarily enriched in *MAF* translocations t(14;16) (30/31, *P* = 1.2 × 10^−15^, mean mutational contribution 0.37) and t(14;20) (7/9, *P* = 4.1 × 10^−3^, mean mutational contribution 0.28) and to a lesser extent with t(4;14) (46/93, *P* = 1.1 × 10^−5^, mean mutational contribution 0.07).

**Fig. 5:**
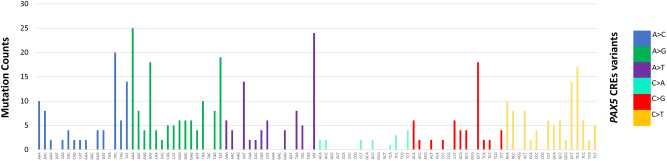
Mutational signatures in multiple myeloma. Mutational patterns of somatic mutations in CREs interacting with *PAX5* promoters display both canonical (C > T/G in WRCY motifs with R = purine, Y = pyrimidine) and non-canonical (A > C/G in WA motifs) activation-induced cytidine deaminase (AID) signatures

Other mutational signatures previously reported in MM [3, 8, 9, 29] were also identified, including signature 1, 5, 9, and 13 in 18% (135/765), 73% (557/765), 96% (737/765), and 5% (36/765) of tumors, respectively (Supplementary Table 16). Almost all samples (35/36) with signature 13 also exhibited signature 2, consistent with the published literature [8]. Mutational signatures not previously reported in MM included signature 3, 8, 16, and 30 seen in >30% of tumors (Supplementary Table 16). No additional signatures were identified when analyzing the high-coverage WES data. Signature 9 (T > G in WT motif with W = A or T), a consequence of activation-induced cytidine deaminase (AID) activity [8], is also a feature of CLL and B-cell lymphomas. The fact that, despite its prevalence, this signature had not previously been identified in earlier large-scale analyses, agrees with the assertion that AID-related mutations are enriched in non-coding regions and early mutation events [29]. As signature 9 suggests AID off-target activity, we examined the mutational patterns of somatic variants affecting the *PAX5* CREs, known off-targets of AID in B-cell malignancies [30]. Somatic mutations in CREs interacting with *PAX5* promoters showed both canonical AID (C > T/G in WRCY motifs with R = purine, Y = pyrimidine) and non-canonical AID (A > C/G in WA motifs) [31] mutational signatures (Fig. 5), in agreement with *PAX5* enhancers mutated by AID in mouse B-cells and diffuse large B-cell lymphoma [30].

## Discussion

This analysis has identified new coding and non-coding drivers, as well as highlighting that pathways, key to the development of MM, can be targeted somatically through a range of mechanisms (Fig. 4). Strikingly, although upregulation of *MYC* through gene amplification or translocation is well established in MM [1], we demonstrate that *MYC* can be dysregulated by alternative mechanisms. These include CNVs altering *MYC* non-coding regulatory regions and specifically, our data implicate a region syntenic to the murine *Myc* enhancer cluster that has recently been reported to be essential for the maintenance of *MLL–AF9*-driven leukemia in mice [32].

The downregulation of tumor suppressors *PAX5* [15–17] and *HOXB3* [23] by CRE mutations in MM is entirely consistent with their decreased expression contributing to development and progression of MM as is the case with other B-cell malignancies. It has previously been demonstrated that disruption of the NF-κB pathway in MM can be the consequence of coding mutations and loss of genes. Here we add *TWEAK*, *TRAF2*, and *PRKD2* to the list of genes disrupted via coding mutations, demonstrate *COBLL1* as dysregulated via mutations of a non-coding regulatory region, and identify *MAP3K14* as upregulated via translocation to the IG loci [33].

Although utilizing WGS data facilitate the identification of signatures enriched in the non-coding genome, it also, by nature of the low-coverage data, focuses the analysis on early mutational processes. Accepting this limitation, we identified a number of mutational signatures previously unreported in MM, and strikingly the AID-attributed signature 9 being detectable in a high proportion of MM, a finding consistent with a contemporaneous report [29]. Although mutational patterns suggestive of AID activity have been documented in certain genes in MM such as *EGR1* [4] and *CCND1* [3], our findings suggest that off-target AID activity could be more widespread than previously envisaged. Moreover, as off-target AID activity is associated with genomic instability and chromosomal translocation in B-cells [34], it may be a major etiological factor driving mutation of MM.

We acknowledge that the present analysis has limitations. First, we have used a cellular model of naïve B-cells to map the CREs, which is unlikely to fully and specifically recapitulate the spectrum of pathogenic SNVs and CNVs seen in MM. Second, the low coverage of CoMMpass WGS data means that we have likely underestimated the somatic variants in the tumors, and increased noise to our gene expression analysis. The sensitivity of our analysis is dependent on the clonal architecture of the samples, and it is likely that our analysis is limited to the identification of clonal, early drivers of MM. Third, inevitably as CNVs are highly recurrent in MM [1], this has restricted the study power of our gene expression analysis as samples were excluded. Finally, non-coding RNAs were not considered in gene expression analysis although many have been identified as recurrently mutated in their regulatory regions (Supplementary Table 2-3). Despite our restricted sensitivity, we have identified multiple targets of non-coding mutations, highlighting the importance of broadening the search for cancer drivers into the regulatory genome. Validation of the candidates we have identified will be contingent on functional studies including, for example, CRISPR-mediated genome editing, in vitro reporter assays, and proliferation assays coupled with transcriptional profiling.

In conclusion, our findings provide integrated analysis of novel coding and non-coding drivers in MM, demonstrating the genetic complexity contributing to this malignancy. Thus by developing a more comprehensive picture of the underlying genetic basis of MM, we extend the list of genes and pathways for which novel therapeutic agents may be identified through network-based drug search methodologies [35, 36], offering the prospect of future individualized therapy in MM.

## Materials and methods

### Sequencing datasets

All data analyzed were generated as part of the Multiple Myeloma Research Foundation (MMRF) CoMMpass Study (release IA9). WGS data on 765 matched tumor-normal baseline newly diagnosed bone marrow samples were downloaded from the database of Genotype and Phenotype (dbGaP). MM tumor specimens were enriched from bone marrow aspirates by CD138 antibody conjugation yielding on average 99% CD138+ plasma tumor cell purity [37]. Matched tumor RNAseq for 606 of the 765 samples, processed by HTseq, were used for gene expression analysis. CNV, WES variants, RNAseq, and sequencing based fluorescent in situ hybridization FISH (Seq-FISH) data (MMRF IA9 dataset) were downloaded from MMRF web portal (https://research.themmrf.org/).

### Significantly mutated coding genes

To identify significantly mutated genes, we annotated WES data using Oncotator [38] and applied MutSigCV [24] (v1.2) adopting default settings (http://www.broadinstitute.org/cancer/cga/mutsig/). Genes with *Q* < 0.05 were considered significantly mutated.

### Genome-wide somatic variant calling

Raw sequencing reads of WGS data were quality checked using FastQC (v.0.11.4, http://www.bioinformatics.babraham.ac.uk/projects/fastqc/), and aligned by the Burrows-Wheeler Alignment tool [39] (BWA v0.7.12) to the human genome hg19/GRCh37 assembly. Mutations in samples were called using MuTect [40] (v1.1.7) according to best practices (https://software.broadinstitute.org/gatk/best-practices/) making use of data from dbSNP v147 and COSMIC non-coding variants v77 [41] to minimize false positives attributable to germline variation. Variants were then filtered for oxidation artifacts [42] and only retained if they had a minimum of one alternative read in each strand direction, a mean Phred base quality score > 26, a mean mapping quality ≥ 50, and an alignability score of 1.0 based on alignability of 75mers defined by the ENCODE/CRG GEM mappability tool [9, 43].

### Assessment of WGS variant calling

We estimated the sensitivity to detect clonal mutations in the low-coverage WGS dataset by comparing called variants with those identified in the high-coverage WES data in IA9 dataset (alternate allele ratio > 0.2).

### Analysis of CNVs

Deletions and amplifications were defined as abs(log_2_ratio) ≥ 0.1613 based on circular binary segmentation defined copy number segments. A chromosome was considered amplified if at least 90% of the chromosome overlapped with an amplification. Cytoband definitions (hg19) were downloaded from UCSC (http://hgdownload.cse.ucsc.edu/goldenpath/hg19/database/). Gene exon boundaries were downloaded from RefSeq (hg19). Affected genes and cytobands were identified by overlaying CNVs using bedtools [44]. Plots were produced using the package karyoploteR [45].

### Analysis of structural variants

BAM files were analyzed and annotated using Illumina’s MANTA [46] and NIRVANA [47] software with default settings, allowing identification of structural variants (SVs) falling within gene boundaries. To search for genes in the vicinity of breakpoints whose expression may be affected by SVs, we first assembled the composite chromosome (as per SAMtools variant call format v4.1 specifications) and then identified genes within 1 Mb of the breakpoints using the RefGene database. The immunoglobulin loci IGH, IGK, and IGL were defined to occur at 14q32.33, 2p11.2, and 22q11.22, respectively. Plots were produced using Circos [48].

### Assignment of myeloma karyotype

Classifications of translocations in MM in MMRF IA9 was based on Seq-FISH [49]. HD was defined as amplification of 90% of the chromosome in at least two autosomal chromosomes. Associations between the number of somatic mutations and MM karyotype were performed using a Wilcoxon rank-sum test comparing the distribution of mutations for each karyotype with all other samples.

### Defining regulatory regions

Promoter regions were defined as intervals spanning 400-bp upstream and 250-bp downstream of the annotated TSS from RefGene database [50] as per Rheinbay et al. [11].

CREs were defined using publicly accessible promoter CHi-C data generated on naïve B-cells [6]. We only considered promoter–CRE interactions with linear distance ≤ 1 Mb [51] and as previously advocated, only interactions with a CHiCAGO score ≥ 5 were considered statistically significant [52].

To remove false positives in regions not identified as duplicates in hg19/GRCh37, we performed an additional filtering step, removing contacts between fragments mapping to regions that did not map to unique locations in hg38. Briefly, hg19/GRCh37 was split into windows of 100 bp prior to alignment to hg38 using BWA. A base was considered to be poorly mapped if the majority of reads containing it could be mapped elsewhere in the genome with at most one mismatch or gap, as described in http://bit.ly/snpable. A contact region was retained if 95% of its constituent bases were well mapped.

### Identification of recurrently mutated regulatory regions

Promoter and CREs were tested independently for recurrence of non-coding mutations based on the approach of Melton et al. [53]. Briefly, the statistical modeling of recurrent mutations assumes a Poisson binomial model, in which the mutation probability for each regulatory region in each tumor is determined by fitting a logistic regression model with glm R function to all data in CREs and promoters separately, taking into account the following factors at every nucleotide base [53]: tumor ID, mutational status, reference base pair (A/T versus G/C), replication timing, and coverage. As replication timing influences mutational rate at each nucleotide base [54], replication timing at a base position was estimated as the average of replication timing data from Hela, K562, HEPG2, MCF7, and SKNSH cell lines [54]. CRE regions that overlap with open reading frames (extended by 5 bp to account for splice sites), and 5’-UTR and 3’-UTR as defined by Ensembl v73 [55] were excluded from the analysis. For promoters, mutations overlapping with open reading frames as defined by Ensembl v73 [55] were excluded.

The mutation probability of each defined regulatory region is defined as:

$${\mathrm{P}}\left( {region\,is\,mutated} \right) = \mathop {\prod }\limits_{k = 1}^s (1 - p_k)$$

where $s$ is the size of the regulatory region tested, $k$ is the nucleotide position, $p_k$ is the mutational probability at base $k$. The Poibin R package was used for approximation of Poisson binomial to estimate the empirical *P*-value for each CRE and promoter regions as per Melton et al. [53].

Mutations in each promoter and CRE region were tested for clustering based on the number of mutations occurring at the same nucleotide positions across all samples in the defined region, as recurrence of exact somatic mutations across different tumor samples implies particular SNVs have an impact on tumorigenesis. For each regulatory region containing at least three mutations [11], the mutation positions were permuted 10,000 times within the same length of the tested region under uniform distribution. The empirical clustering *P*-value for each tested region was calculated as the fraction of times that a set of permutated mutations having at least the same number of mutations occurring at the exact position as in the tested region.

The clustering *P*-value and background estimated *P*-value were combined, implementing the Fisher method within metap R package to derive combined *P*-values for recurrent mutation as per Rheinbay et al. [11]. The Benjamini–Hochberg false discovery rate (FDR) procedure was used to adjust for multiple-hypothesis testing with significance thresholded at *Q* < 0.05.

### Effect of regulatory region SNVs on gene expression

Promoter and CRE regions, which were significantly mutated, were examined for differential gene expression. We tested for a difference in gene expression between mutated and non-mutated tumors using a negative binomial model [55], implemented in edgeR [56]. Samples with CNVs (including aneuploidy) at either the gene or the related regulatory regions were excluded [55]. Regulatory regions were not tested if the CRE was mutated in fewer than three samples, after the removal of samples with overlapping CNVs. Where many mutated CREs were identified as interacting with a promoter, tumors harboring mutations in more than one CRE fragment were excluded and only samples with no mutations in any of the recurrently mutated CREs were used for comparison. Regulatory regions interacting with multiple genes were tested multiple times. Only CREs interacting with protein-coding genes were evaluated. *P*-values obtained were adjusted by Benjamini–Hochberg FDR. Regions with fold change in gene expression ≥ 1.2 or ≤ 0.8, and threshold *Q* < 0.1 are reported.

### Epigenetic annotation of CREs

Naive B-cell ChIP-seq data for H3K4me1, H3K4me3, H3K27me3, and H3K27ac marks (sample EGAN00001265744-S00XAQH1) were downloaded from BLUEPRINT. UCSC LiftOver tool (http://genome.ucsc.edu/cgi-bin/hgLiftOver) was used to derive genome coordinates.

### Gene-set enrichment analysis

Gene ontology (GO) term enrichment analysis was performed to examine for the over-representation of sets of genes for specific GO annotations. To ensure that the analysis was not biased toward GO term annotations enriched among genes whose promoters interact with greater numbers of CREs, we annotated the individual CRE–promoter interactions with the GO terms associated with the contacted genes, and completed the enrichment analysis at the level of the CRE–promoter interaction for CREs and all TSS defined for a gene, rather than the gene level. Hence, all promoters and CRE–promoter interactions were used as the background set. Enrichment of GO term annotations obtained from GO.db [57] were tested using a hypergeometric test. The 37 GO terms spanning 10 previously defined cancer hallmarks [58] and in signaling pathways involved MM, including NIK/NF-κB signaling, MAPK signaling, B-cell proliferation, and B-cell activation and differentiation were tested.

### Analysis of gene expression and CNVs at CREs

Focal deletions and amplifications by CNVs were defined as abs(log_2_ratio) ≥ 0.1613 and size < 3 Mb. We identified tumors with deleted or amplified CREs as those overlapping CNVs and for each promoter-gene, we excluded CREs with (i) amplification or deletion of the target gene (abs(log_2_ratio) ≥ 0.1613); (ii) <7 observations (representing 1% of sample size). We compared gene expression between mutated and unmutated samples using edgeR [56] using default parameters as per SNV analysis.

### Subgroup analysis

We restricted subgroup analysis to the main groups for which we had reasonable power to detect a relationship. Specifically, we included the most frequent myeloma subtypes—HD, t(4:14), t(11:14), and t(14:16)—along with the t(8:14) *MYC* translocation subgroup. For the analysis of coding regions, we assessed the frequency of commonly mutated genes (defined by our analysis and previously published work [2–4]) assessing enrichment based on a Fisher’s exact test. For those CREs we identified as recurrently mutated and differentially expressed, we also compared the frequency of CRE mutation by subgroup assessing enrichment using a Fisher’s exact test. Furthermore, to confirm our combined analysis had not missed any subgroup specific effects, we performed coding and non-coding SNV analyses separately for each subgroup.

### Integrated pathway analysis

We used the Reactome tool [28] to evaluate pathways significantly altered by coding and non-coding drivers identified, with *Q*-values < 0.05 being considered statistically significant.

### Analysis of mutational signatures

All somatic variants from WES and WGS passing filtering were considered for mutational signature analysis. Assignment to the 30 mutational signatures proposed by the Wellcome Trust Sanger Institute (http://cancer.sanger.ac.uk/cosmic/signatures) was performed using the R package deconstructSigs with default parameters [59]. Non-coding variants disrupting CREs corresponding to *PAX5* were analyzed. Associations between APOBEC mutations and MM translocation subgroups, as well as recurrently mutated genes and regulatory regions identified as statistically altering gene expression, were performed using Fisher’s exact test. A *P* < 0.05 (one-sided) was considered statistically significant.

### Data availability

CHi-C data were obtained from Javierre et al. [6]. Histone ChIP-seq sequencing data were downloaded from BLUEPRINT under accession number EGAD00001002466, sample S00XAQH1.

WGS and WES raw fastq data were obtained from dbGaP under the study accession code phs000748.v4.p3.

WES somatic variants, RNAseq, CNV, and Seq-FISH data were obtained from MMRF IA9 (https://research.themmrf.org/).

Replication timing data were downloaded from the UCSC Genome Browser (http://hgdownload.cse.ucsc.edu/goldenPath/hg19/encodeDCC/wgEncodeUwRepliSeq/).

## Electronic supplementary material

Supplementary Note

Supplementary Figures

Dataset 1
