# Supplementary material for: Whole-genome sequencing of multiple myeloma reveals oncogenic pathways are targeted somatically through multiple mechanisms
Source: Leukemia. 2018 Apr 9;32(11):2459–70. doi: 10.1038/s41375-018-0103-3 (PMC6224406; doi:10.1038/s41375-018-0103-3)
Supplement: Supplementary file 1 — Supplementary Note [file 41375_2018_103_MOESM1_ESM.docx]

**Supplementary Note**

**Chromosomal copy number alterations**

Multiple frequent copy number alterations were detected in MM tumors (**Supplementary Fig. 5**). Pre-eminently, gain of odd numbered chromosomes, characteristic of hyperdiploid MM (HD)^1^, was seen in 59% of tumors, with chromosome 9, 15, and 19 most often amplified (83-86% HD, **Supplementary Table 9a**); concordant with published observations^1^. Deletion of chromosomal cytobands containing immunoglobulin (IG) loci IGK (2p11.2), IGH (14q32.33) and IGL (22q11.22) were present in 95%, 98% and 57% of tumors respectively (**Supplementary Fig. 5**, **Supplementary Table 9b**), consistent with the rearrangements expected at IG loci during normal B-cell development^2^. Common deletions were also seen at 13q (63%), 14q (43%), 16q (38%) and 8p (38%). Despite the relatively low overall level of chromosome 8 amplification, 28% of tumors exhibited amplification overlapping 8q24.21 that incorporates *MYC* (13%) and *PVT1* (16%)^3, 4^.

**Structural variation**

The median rate of structural variants (SVs) was 10 across tumors; four translocations (range 0-147) and six inversions (range 0-2,790). Considering SVs falling within gene boundaries, on average six genes were disrupted per tumor. SVs were also identified as affecting genes commonly mutated in MM^5-7^ including *CYLD* with inversions disrupting the protein sequence in five samples (**Supplementary Table 10)**. Widening the definition of SVs to genes within a 1 Mb window of translocation breakpoints identified multiple recurrent rearrangements including *MYC*, *CCND1* and *FGFR3,* detected in 173 (23%), 124 (16%) and 46 (6%) of samples, respectively. *MYC* rearrangements involved a plethora of partner sites including IGH (32/765), IGL (32/765), IGK (11/765), and cytobands encompassing *BMP6* (21/765), *FAM46C* (9/765), *CCND1* (1/765) and *MAF* (1/765). Novel *MYC* translocations disrupting *CD96* (immune checkpoint receptor target) were identified in eight tumors and translocations intergenic to *PRDM1* and *FBXW7* in eight and five tumors, respectively. Restricting this analysis to translocations incorporating the IGH, IGK and IGL loci, we identified common translocations affecting 17q21.31, encompassing *MAP3K14*, in 16 tumors, and 10 tumors with translocations affecting 12p13.32, encompassing *CCND2* (**Supplementary** **Fig. 6**). Tumors with these translocations were associated with upregulation of *MAP3K14* (7.4-fold upregulation, *P* = 5.05 × 10^-41^), and *CCND2* (11.9-fold upregulation,*P* = 7.5 × 10^-5^).

**Significantly mutated protein-coding genes**

To gain insight into mutations affecting protein-coding regions, we applied MutSigCV^8^ to variants identified from WES data. We identified 33 significantly mutated genes (*Q* < 0.05, **Supplementary** **Table 11**). These were over-represented in pathways involved in sustaining proliferative signaling, activating invasion, evading growth suppressors, tumor-promoting inflammation, resisting cell death, enabling replicative immortality, and angiogenesis (*P* < 0.05, **Supplementary Table 12**). While 16 of the 33 genes have previously been documented to be recurrently mutated in MM (*KRAS*, *NRAS, HIST1H1E*, *MAX*, *SP140*, *RASA2, FCF1*, *DIS3, BRAF, TP53*, *SAMHD1*, *TRAF3, PRKD2*, *TGDS*, *CYLD*, and *RB1;* **Supplementary Table 13**)^1, 5-7, 9^[4] we identified 17 novel significantly mutated genes. These included 12 genes previously reported as recurrently mutated, albeit not significantly (*PTPN11*, *DNAH5*, *MYH2*, *BMP2K*, *ZNF208*, *RPL10*, *FBXO4*, *OR5M1*, *PTH2*, *CELA1*, *OR9G1*, and *TNFSF12*)^5-7, 10-12^ and five novel genes (*TBC1D29*, *RPS3A*, *BAX*, *C8orf86*, and *FTL*) (**Supplementary Table 11**).

Stratifying MM according to its major subgroups (HD, *MYC-*translocation, t(4;14), t(11;14), t(14;16)) allowed us to identify additional drivers; *FAM154B*, *HIST1H4H*, *LEMD2* and *PABPC1* in HD; *RPN1* and *TRAF2* in *MYC-*translocation; *SGPP1* in t(11;14); and *TRAF2* in t(14;16) (**Supplementary Table 14**). Furthermore, we identified t(4;14) MM as being enriched for *PRKD2* mutations (13% of subtype, *P* = 1.0 × 10^-5^) but having a paucity of *NRAS* mutations (*P* = 1.3 × 10^-6^); possibly reflecting dysregulation of the MAPK-signaling, a consequence of the translocation-mediated *FGFR3* overexpression (**Supplementary Table 7**). As previously reported, we identified t(11:14) MM, associated with *CCND1* mutation^13^ (10%, *P* = 1.2 × 10^-10^) and *IRF4* mutation (8%, *P* = 8.0 × 10^-6^). In contrast, mutations in *PRKD2* (*P* = 2.0 × 10^-4^), *MAX* (*P* = 1.3 × 10^-6^) and *DIS3* (*P* = 1.6 × 10^-6^) were infrequent in HD. Finally we noted that somatic mutations in the following genes had low alternative allelic fraction - *RPS3A* (range 0.1–0.5), *TBC1D29* (range 0.1–0.5), *PABPC1* (range 0.1–0.4), and *TRAF2* (range 0.1–0.9), reflecting the heterogeneity of MM.

**References**

1. Manier S, Salem KZ, Park J, Landau DA, Getz G, Ghobrial IM. Genomic complexity of multiple myeloma and its clinical implications. *Nat Rev Clin Oncol* 2017 Feb; **14**(2)**:** 100-113.

2. Max EE FS. *Immunoglobulins: molecular genetics*. Philidelphia: Lippincott Williams & Wilkins, 2013.

3. Walker BA, Wardell CP, Brioli A, Boyle E, Kaiser MF, Begum DB*, et al.* Translocations at 8q24 juxtapose MYC with genes that harbor superenhancers resulting in overexpression and poor prognosis in myeloma patients. *Blood Cancer J* 2014 Mar 14; **4:** e191.

4. Nagoshi H, Taki T, Hanamura I, Nitta M, Otsuki T, Nishida K*, et al.* Frequent PVT1 rearrangement and novel chimeric genes PVT1-NBEA and PVT1-WWOX occur in multiple myeloma with 8q24 abnormality. *Cancer Res* 2012 Oct 01; **72**(19)**:** 4954-4962.

5. Walker BA, Boyle EM, Wardell CP, Murison A, Begum DB, Dahir NM*, et al.* Mutational Spectrum, Copy Number Changes, and Outcome: Results of a Sequencing Study of Patients With Newly Diagnosed Myeloma. *J Clin Oncol* 2015 Nov 20; **33**(33)**:** 3911-3920.

6. Lohr JG, Stojanov P, Carter SL, Cruz-Gordillo P, Lawrence MS, Auclair D*, et al.* Widespread genetic heterogeneity in multiple myeloma: implications for targeted therapy. *Cancer Cell* 2014 Jan 13; **25**(1)**:** 91-101.

7. Bolli N, Avet-Loiseau H, Wedge DC, Van Loo P, Alexandrov LB, Martincorena I*, et al.* Heterogeneity of genomic evolution and mutational profiles in multiple myeloma. *Nat Commun* 2014; **5:** 2997.

8. Lawrence MS, Stojanov P, Polak P, Kryukov GV, Cibulskis K, Sivachenko A*, et al.* Mutational heterogeneity in cancer and the search for new cancer-associated genes. *Nature* 2013 Jul 11; **499**(7457)**:** 214-218.

9. Keats JJ, Speyer G, Christofferson A, Legendre C, Aldrich J, Russell M*, et al.* Molecular Predictors of Outcome and Drug Response in Multiple Myeloma: An Interim Analysis of the Mmrf CoMMpass Study. *Blood* 2016; **128**(22)**:** 194-194.

10. Walker BA, Wardell CP, Melchor L, Hulkki S, Potter NE, Johnson DC*, et al.* Intraclonal heterogeneity and distinct molecular mechanisms characterize the development of t(4;14) and t(11;14) myeloma. *Blood* 2012 Aug 02; **120**(5)**:** 1077-1086.

11. Kortum KM, Mai EK, Hanafiah NH, Shi CX, Zhu YX, Bruins L*, et al.* Targeted sequencing of refractory myeloma reveals a high incidence of mutations in CRBN and Ras pathway genes. *Blood* 2016 Sep 01; **128**(9)**:** 1226-1233.

12. Hofman IJF, Patchett S, van Duin M, Geerdens E, Verbeeck J, Michaux L*, et al.* Low frequency mutations in ribosomal proteins RPL10 and RPL5 in multiple myeloma. *Haematologica* 2017 Aug; **102**(8)**:** e317-e320.

13. Walker BA, Wardell CP, Murison A, Boyle EM, Begum DB, Dahir NM*, et al.* APOBEC family mutational signatures are associated with poor prognosis translocations in multiple myeloma. *Nat Commun* 2015 Apr 23; **6:** 6997.
