# Supplementary material for: Whole-genome sequencing of multiple myeloma reveals oncogenic pathways are targeted somatically through multiple mechanisms
Source: Leukemia. 2018 Apr 9;32(11):2459–70. doi: 10.1038/s41375-018-0103-3 (PMC6224406; doi:10.1038/s41375-018-0103-3)
Supplement: Supplementary file 2 — Supplementary Figures [file 41375_2018_103_MOESM2_ESM.docx]

**SUPPLEMENTARY FIGURES**

**
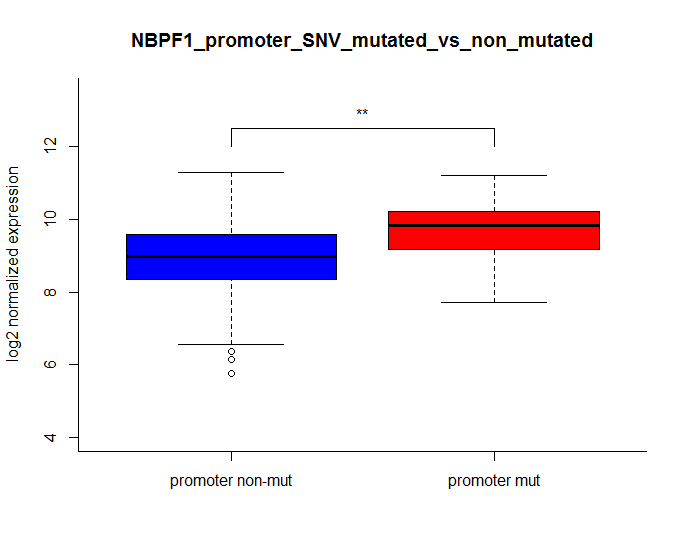
**

**Supplementary Figure 1.** Mutations in the promoter region affect gene expression of *NBPF1*. (*n* = 461 versus *n* = 14). ** *Q* < 0.05. The hinges of the boxplot indicate the first and third quartile range.


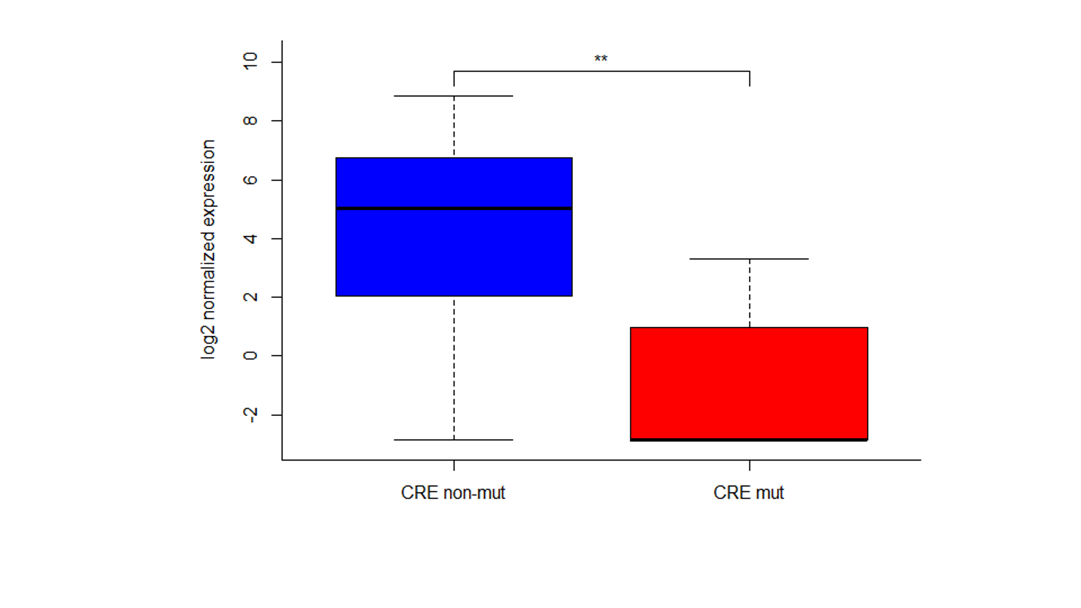

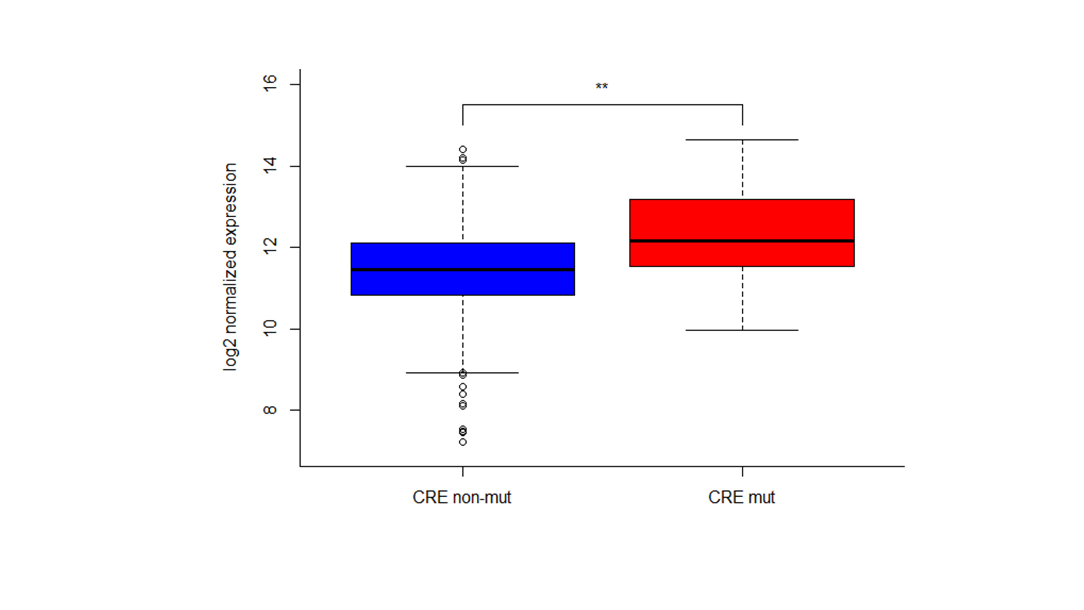


d

c

b

a


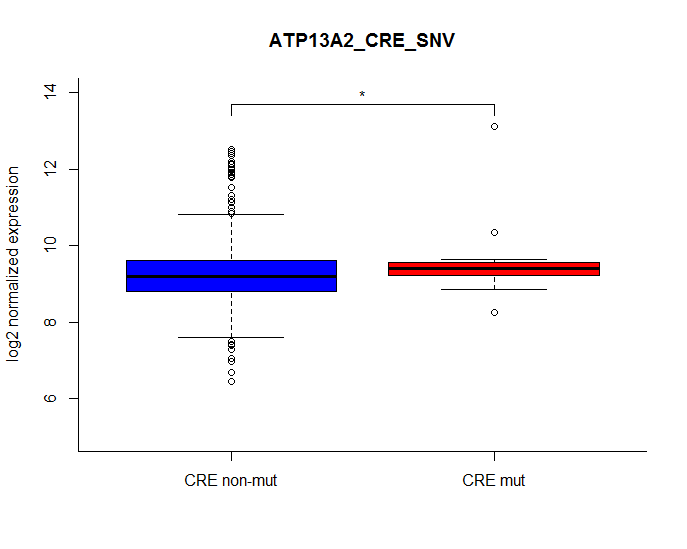

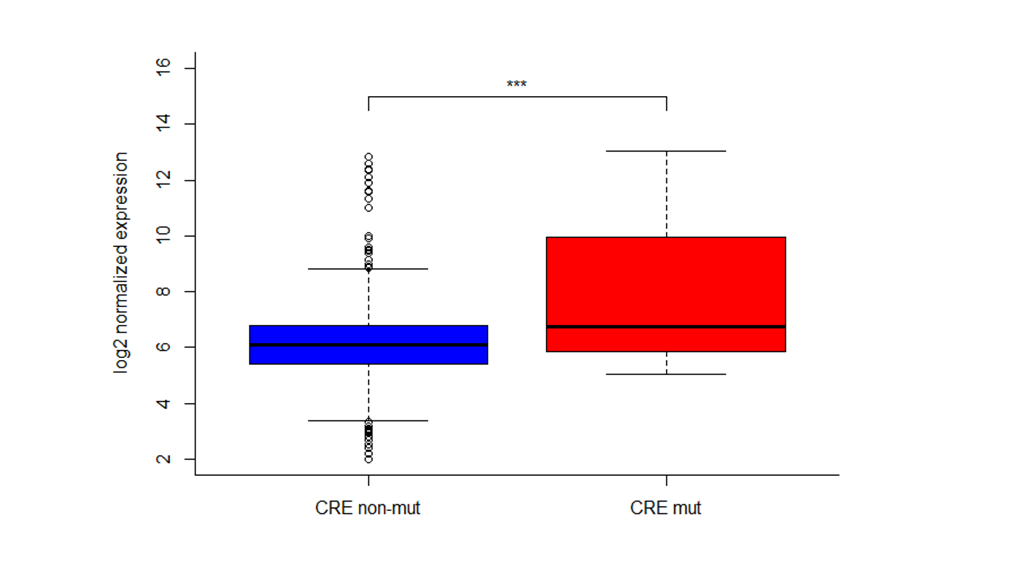


**Supplementary Figure 2.** *Cis*-regulatory element mutations affect gene expression of (**a**) *COBLL1* (*n* = 491 versus *n* = 8), (**b**) *HOXB3* (*n* = 453 versus *n* = 5), (**c**) *CALCB* (*n* = 365 versus *n* = 4) and (**d**) *ATP13A2* (*n* = 461 versus *n* = 14). * *Q* < 0.1, ** *Q* < 0.05, *** *Q* < 0.01. The hinges of the boxplot indicate the first and third quartile range.


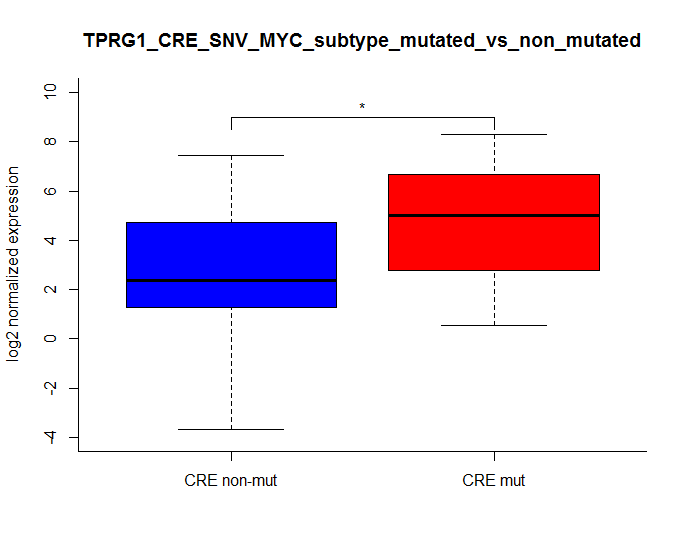


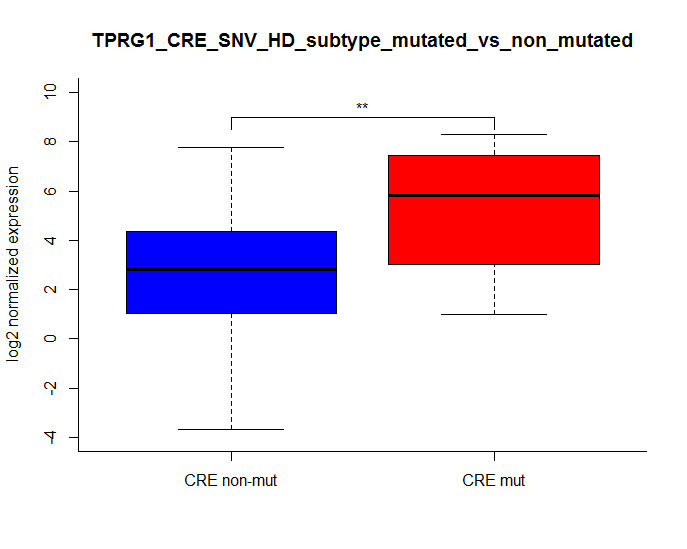


b

a

**Supplementary Figure 3.** *Cis*-regulatory element mutations affect gene expression of *TPRG1* in (**a**) HD subtype (*n* = 114 versus *n* = 4) and (**b**) MYC subtype (*n* = 31 versus *n* = 3). * *Q* < 0.1, ** *Q* < 0.05. The hinges of the boxplot indicate the first and third quartile range.

b

a

c

d

c


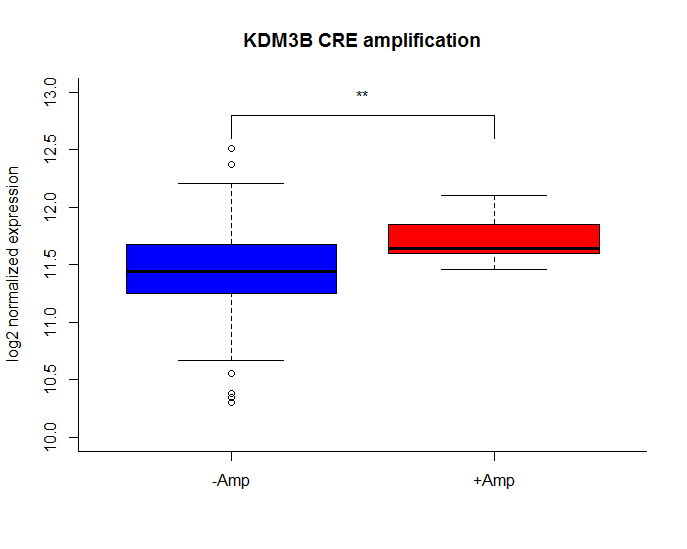

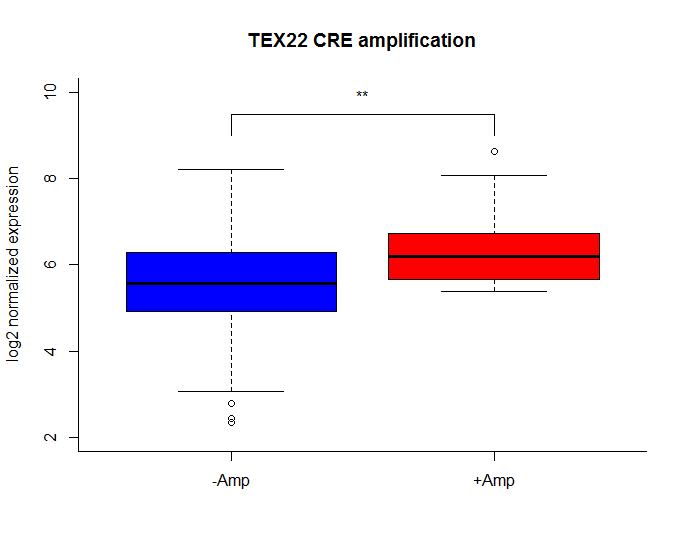

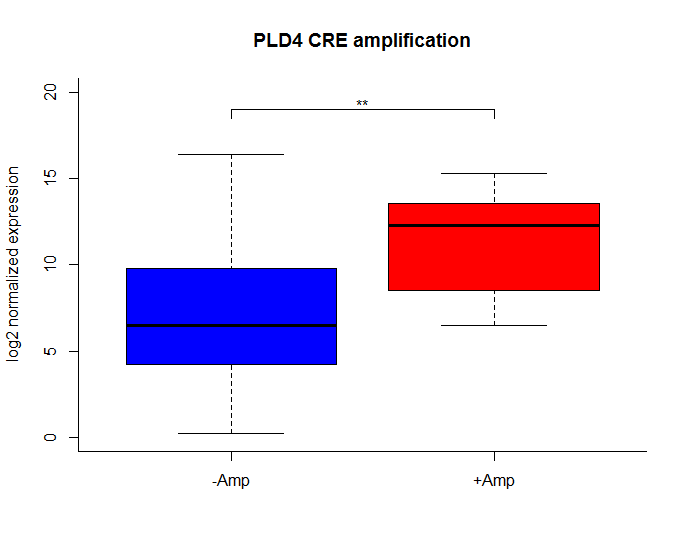


f

e

a

c

e

a


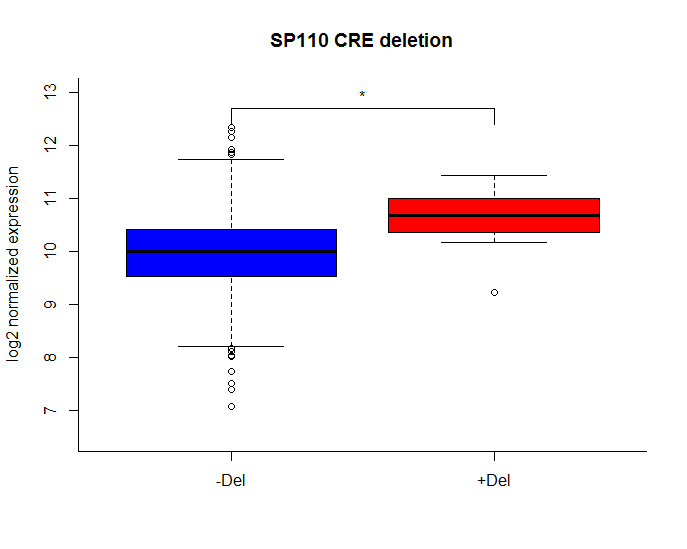


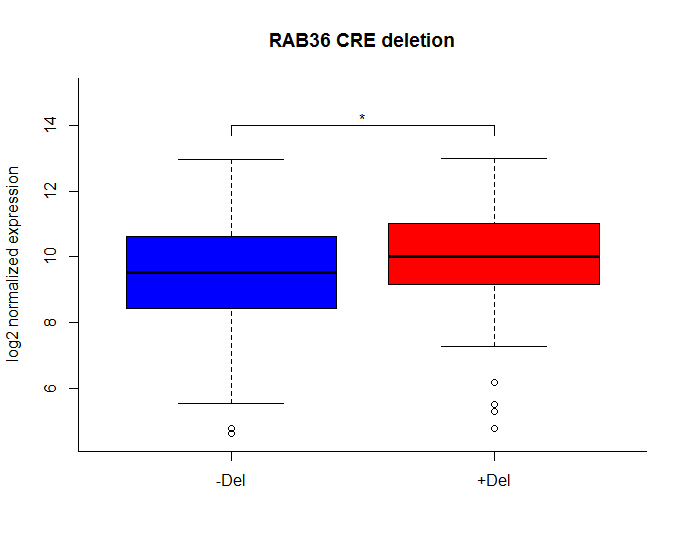

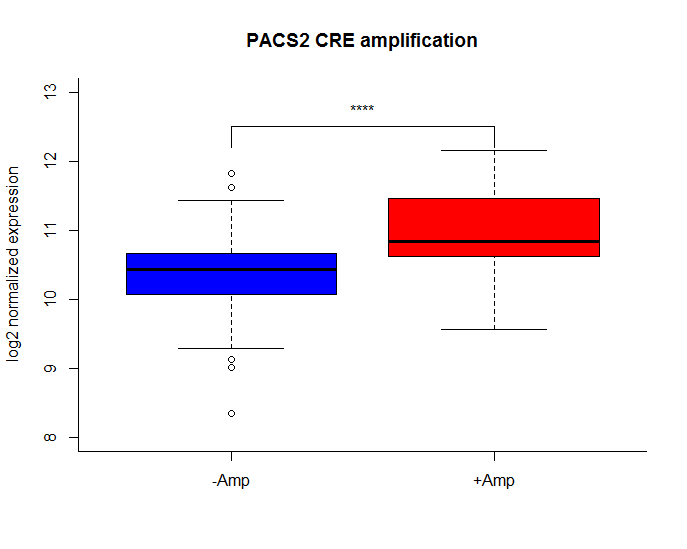
**Supplementary Figure 4. The effects of CNVs at CREs on gene expression in MM.** Boxplots show differential gene expression between CNV unaffected (blue) versus CNV affected samples (red) at CREs interacting with promoters of (a) *PACS2* (*n* = 333 versus *n* = 21); (b) *TEX22* (*n* = 333 versus *n* = 12); (*c*) *PLD4* (*n* = 352 versus *n* = 8); (d) *KDM3B* (*n* = 285 versus *n* = 9); (e) *RAB36* (*n* = 236 versus *n* = 133); and (f) *SP110* (*n* = 462 versus *n* = 7). * *Q* < 0.1, ** Q < 0.05, **** Q < 0.001. Amp, amplification. Del, deletion. The hinges of the boxplot indicate the first and third quartile range.**
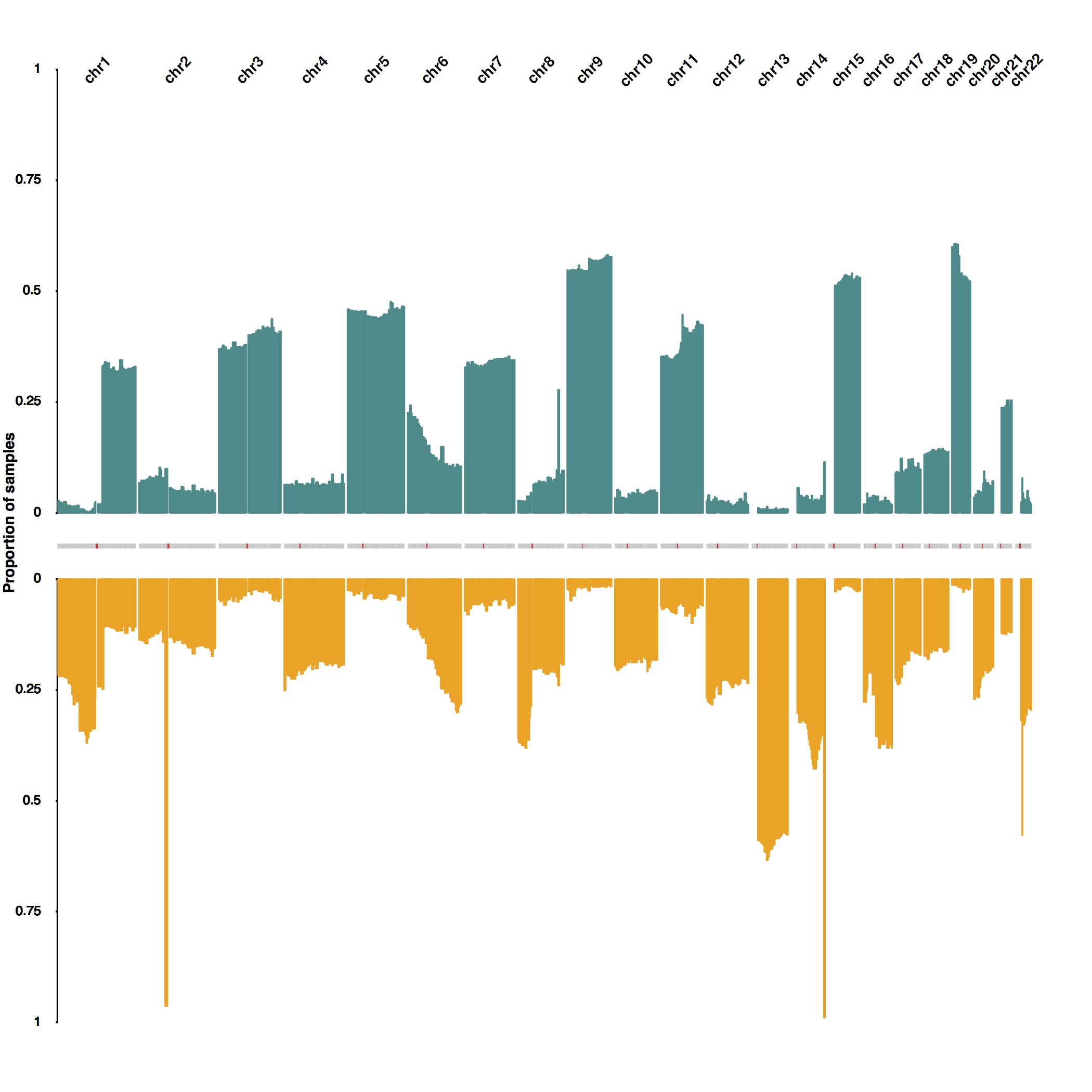
Supplementary Figure 5. Summary of amplifications and deletions in 725 MM samples.** The proportion of samples with amplifications (cyan) and deletions (orange) overlapping each cytoband is plotted by karyoplotR^57^. The frequent deletions (orange peaks) at 2p11.2, 14q32.33 and 22q11.22 overlap with the immunoglobulin loci IGK, IGH, and IGL respectively.

a


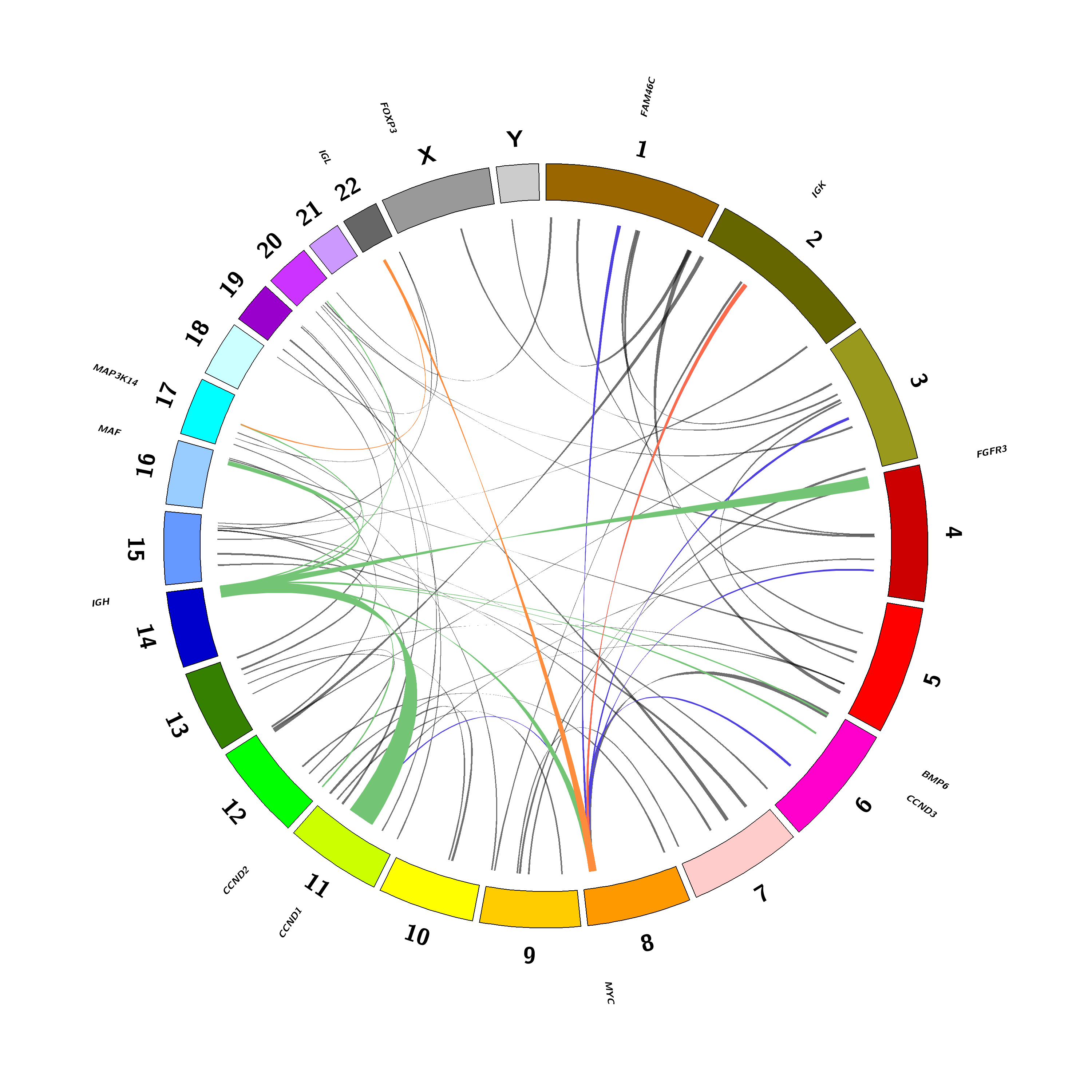


**Supplementary Figure 6. Circos plot of common translocations (> 5 samples)**. IGK (chr2) IGH (chr14) and IGL (chr22) translocations are depicted in red, green and orange, respectively. *MYC* translocations in blue. The ribbon is centered on the cytoband implicated with the ribbon width proportional to the number of affected samples.
